# Supplementary figures and images for: The sialidase NEU3 promotes pulmonary fibrosis in mice
Source: Respir Res. 2022 Aug 23;23:215. doi: 10.1186/s12931-022-02146-y (PMC9400331; doi:10.1186/s12931-022-02146-y)

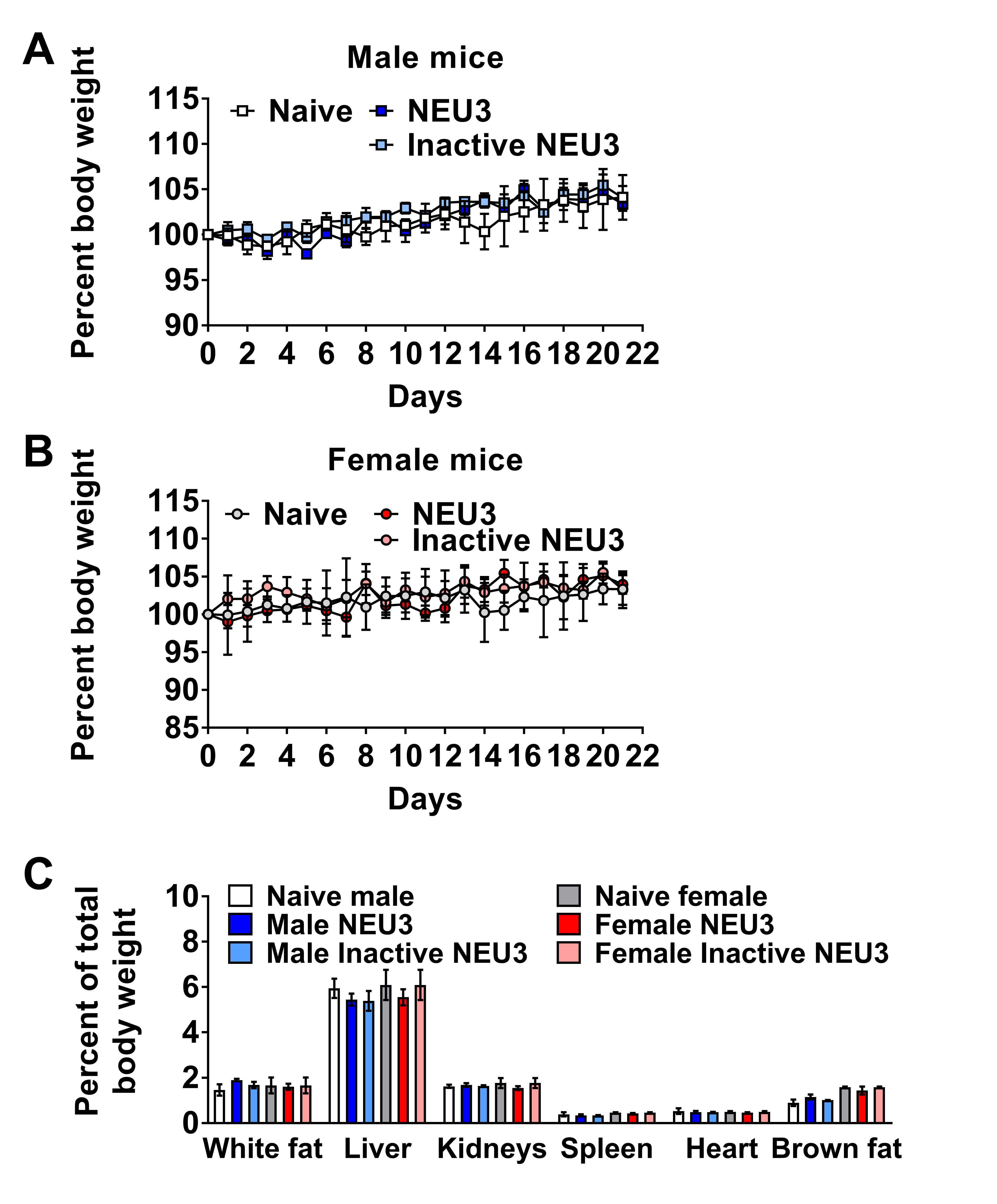

Supplement: Supplementary file 1 — Additional file 1: Fig S1. Aspiration of recombinant murine NEU3 or inactive NEU3 had no significant effect on body weight or organ weights. Percent change in body weight of A) male and B) female naïve mice, or mice after aspiration of recombinant (rec) murine NEU3 or inactive NEU3 for every 48 h for 20 days. C) Weights of white fat, liver, kidneys, spleen, heart, and brown fat as percentage of total bodyweight at day 21. Values are mean ± SEM, n = 6 (3 male and 3 female mice). There were no significant changes as determined by t test or one-way ANOVA, Bonferroni’s or Sidak’s test. [file 12931_2022_2146_MOESM1_ESM.tif]

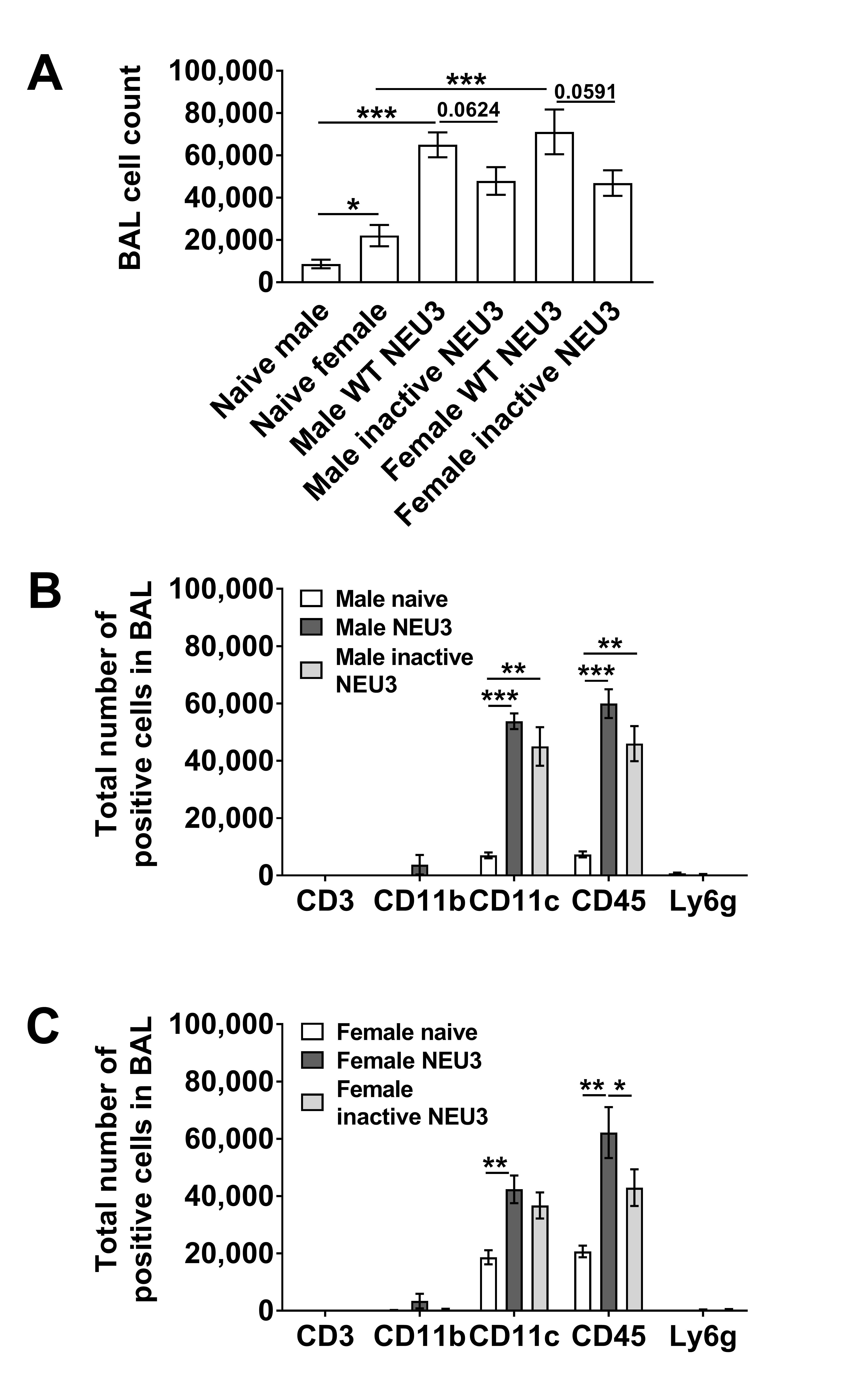

Supplement: Supplementary file 2 — Additional file 2: Fig S2. NEU3 treated male and female mice have increased numbers of bronchoalveolar lavage (BAL) cells. A) The total number of cells in mouse BAL after the indicated treatment from male and female mice. Values are mean ± SEM, n = 3 male and 3 female mice per group. B-C) BAL cell spots at day 21 were stained for the markers CD3, CD11b, CD11c, CD45, and Ly6g, and the percent of cells stained was determined, and the percentage was multiplied by the total number of BAL cells for that mouse to obtain the total number of BAL cells staining for the marker. Values are mean ± SEM., n = 3 male and 3 female mice. *p < 0.05; **p < 0.01, *** p < 0.001 (one-way ANOVA, Bonferroni’s or Sidak’s’s test). [file 12931_2022_2146_MOESM2_ESM.tif]

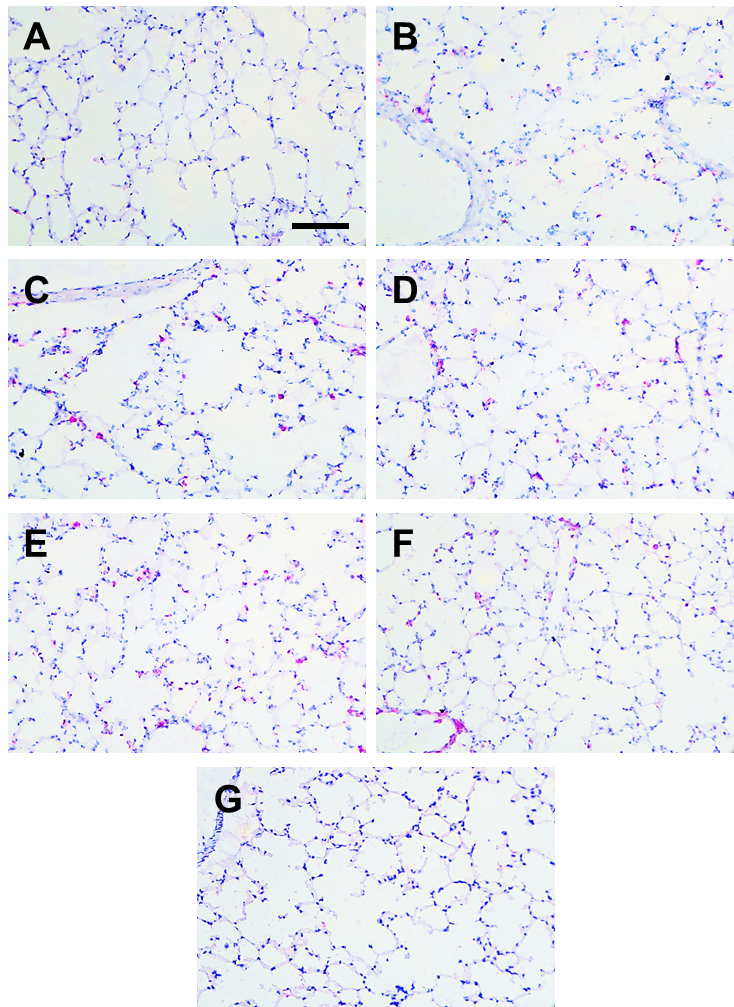

Supplement: Supplementary file 3 — Additional file 3: Fig S3. Increase in CD45 positive immune cells in lungs post-BAL of NEU3 treated male and female mice. Cryosections of male and female mouse lungs were stained for CD45. A and B) Naïve (control), C and D) NEU3 (WT), and E and F) inactive NEU3 treated male A, C, E), and female B, D, and F) mice. G) Isotype irrelevant control antibody. All images are representative of three mice per group. Bar is 0.1 mm. [file 12931_2022_2146_MOESM3_ESM.tif]

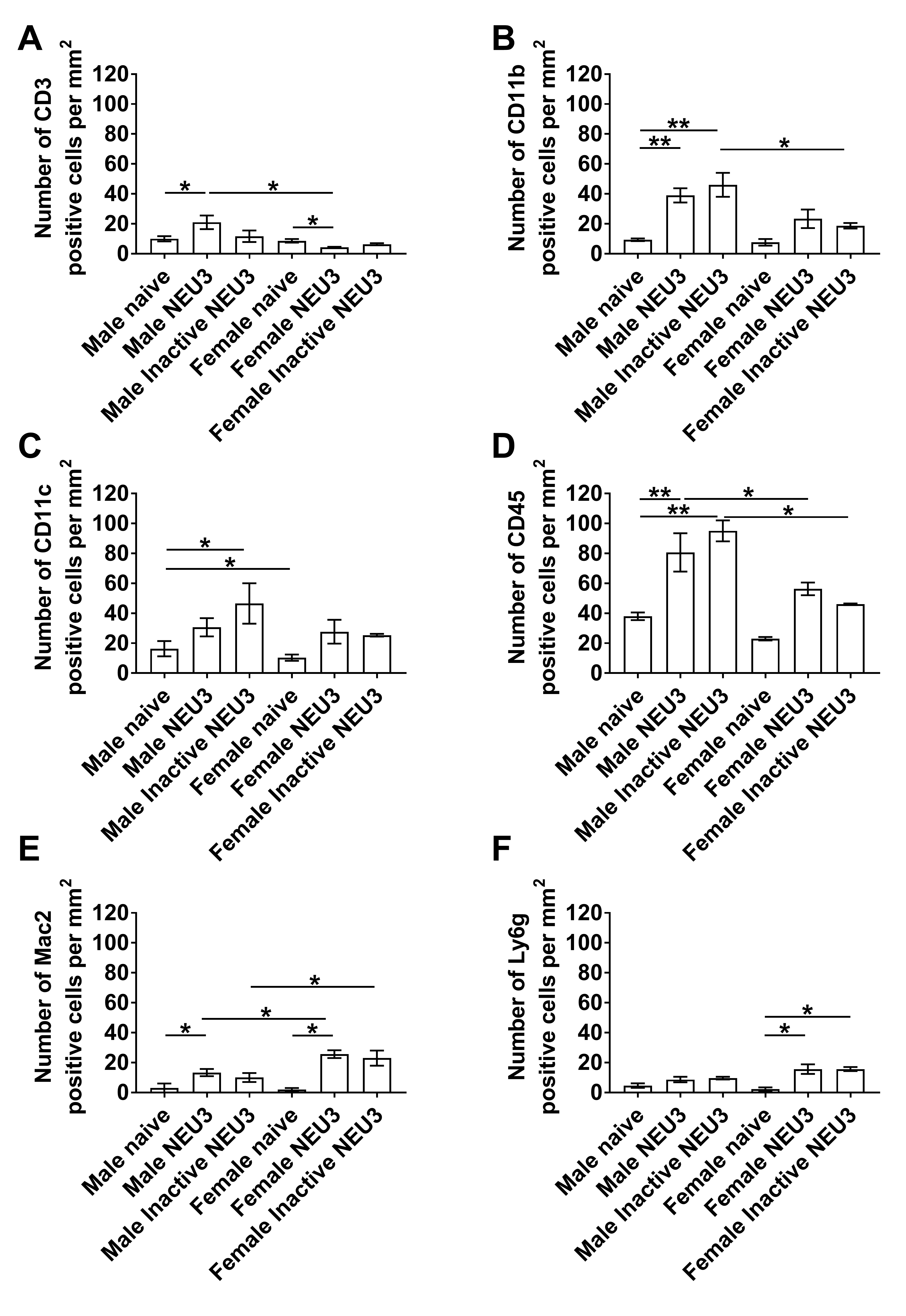

Supplement: Supplementary file 4 — Additional file 4: Fig S4. Increase in immune cells in lungs post-BAL of NEU3 treated male and female mice. Cryosections of male and female mouse lungs were stained for A) CD3 B) CD11b, C) CD11c, D) CD45, E) Mac2 and F) Ly6g and counts were converted to the number of positive cells per mm2. Values are mean ± SEM, n = 3 male and 3 female mice. *p < 0.05; ** p < 0.01 (one-way ANOVA, Bonferroni’s or Sidak’s test). [file 12931_2022_2146_MOESM4_ESM.tif]

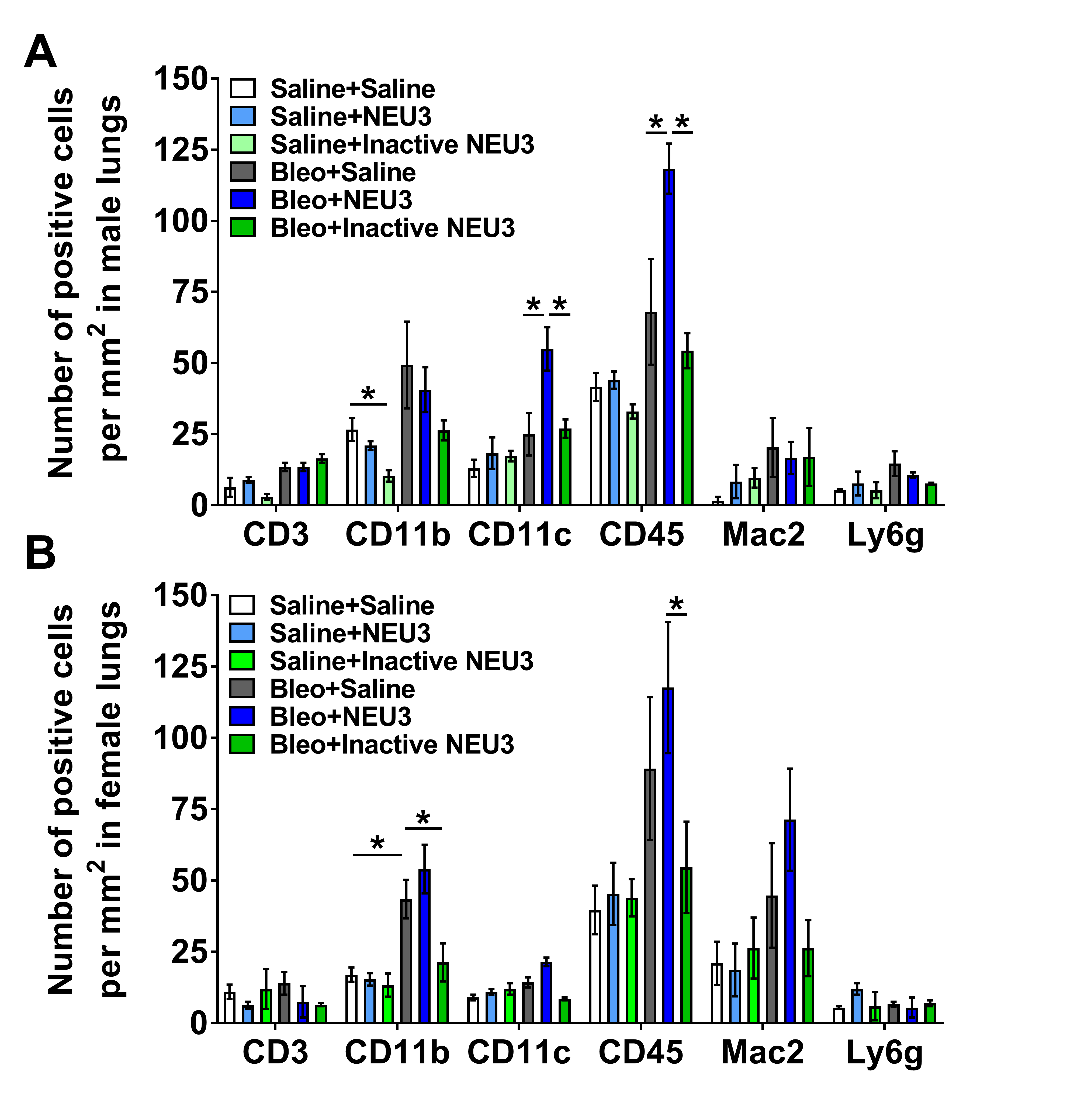

Supplement: Supplementary file 5 — Additional file 5: Fig S5. NEU3, but not inactive NEU3, treated male and female mice have increased numbers of inflammatory cells in lung tissue following bleomycin aspiration. Cryosections of A) male and B) female mouse lungs were stained for CD3, CD11b, CD11c, CD45, Mac2, and Ly6g, and counts were converted to the number of positive cells per mm2. Values are mean ± SEM, n = 3 male and 3 female mice. *p < 0.05; (one-way ANOVA, Bonferroni’s or Sidak’s test). [file 12931_2022_2146_MOESM5_ESM.tif]

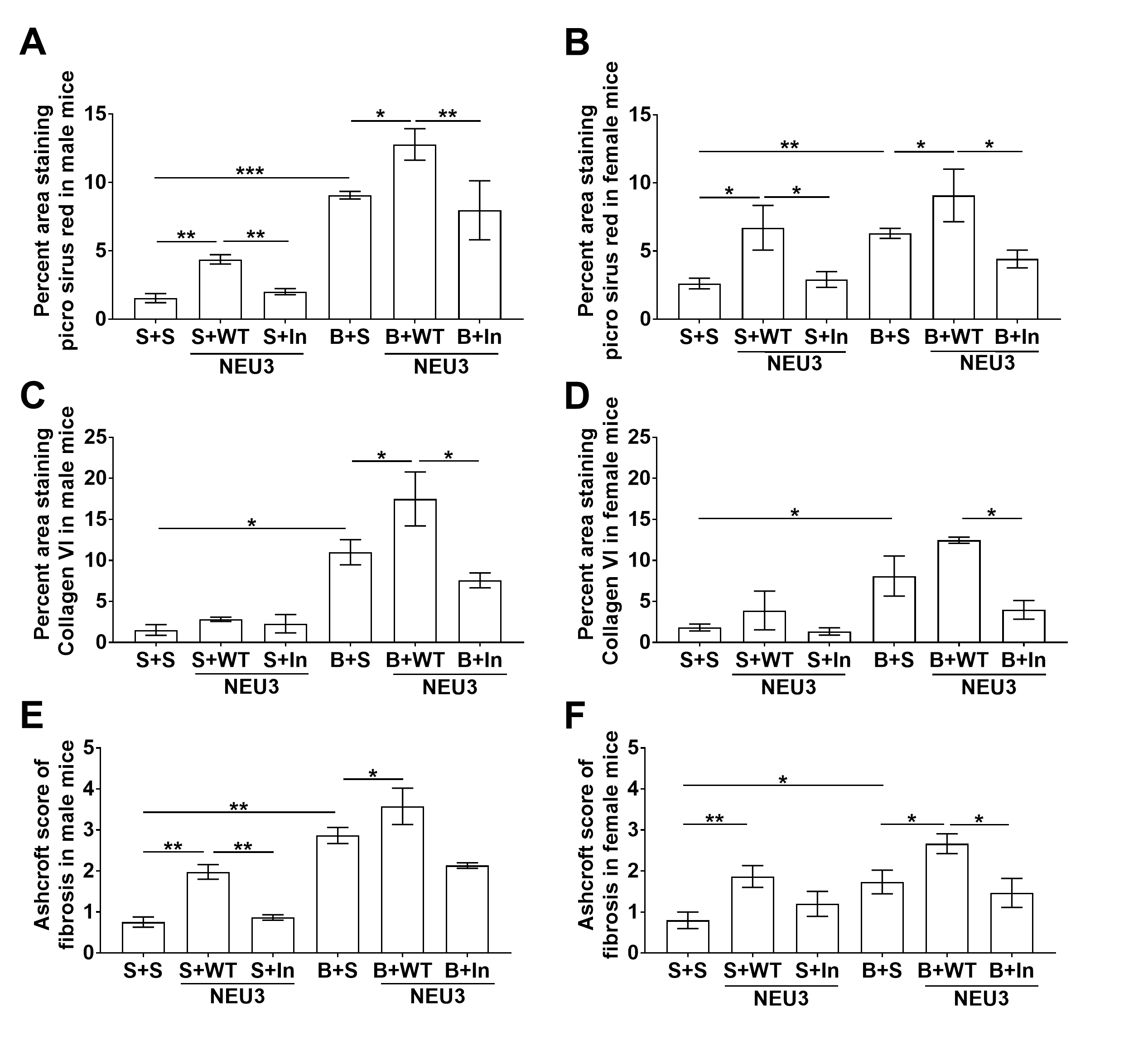

Supplement: Supplementary file 6 — Additional file 6: Fig S6. Enhanced fibrotic response in NEU3 treated male and female mice following bleomycin. Sections of lung tissue from male and female mice treated with saline (S) or bleomycin (B) aspiration at day 0, and NEU3 (WT) or inactive (In) NEU3 treatment from days 10 to 20. Quantification of A-B) picrosirius red, C-D) anti-collagen VI antibody staining, and E–F) histological assessment of fibrosis. Values are mean ± SEM, n = 3 male and n = 3 female mice. *p < 0.05; **p < 0.01 ***p < 0.001 (one-way ANOVA, Bonferroni’s or Sidak’s test). [file 12931_2022_2146_MOESM6_ESM.tif]
